# EEG changes associated with hallucinations caused by Charles Bonnet Syndrome

**DOI:** 10.3389/fneur.2025.1697094

**Published:** 2026-01-06

**Authors:** Jasleen K. Jolly, Natalie Assaf, Bethany E. Higgins, Jane E. Aspell, Elizabeth Michael

**Affiliations:** 1 Jolly Vision Science, Cambridge, United Kingdom; 2 Faculty of Health, Medicine and Social Care, Vision and Eye Research Institute, Anglia Ruskin University, Cambridge, United Kingdom; 3 Department of Optometry and Vision Science, Melbourne University, Melbourne, VIC, Australia; 4 Department of Optometry and Visual Sciences, City St George’s, University of London, London, United Kingdom; 5 School of Psychology, Sport and Sensory Sciences, Faculty of Science & Engineering, Anglia Ruskin University, Cambridge, United Kingdom; 6 MRC Cognition & Brain Sciences Unit, University of Cambridge, Cambridge, United Kingdom

**Keywords:** CBS, alpha waves, visual brain, electroencephalography, visual impairment

## Abstract

Charles Bonnet syndrome (CBS) is characterised by the presence of visual hallucinations following visual loss in many patients. The neuropathophysiology of CBS is poorly understood. We used electroencephalography (EEG) in individuals with frequent hallucinations in order to identify changes in neural activity that co-occur with hallucination onset. We found reduced α power in occipital electrodes at the onset of the hallucinations compared to offset (*p* < 0.05), suggesting that intermittent periods of low endogenous alpha may create the neural conditions for hallucination to emerge in CBS patients. This is the first group study that shows a repeatable marker of brain activity changes in CBS that occur either at the onset or just prior to the onset of the hallucination. This offers important implications for both research and clinical practice. It could aid in early detection and prediction of hallucination onset and improve our understanding of the neural mechanisms underlying CBS. It also may help reduce stigma around the condition by validating the patient experience through measurable brain changes.

## 1 Introduction

Charles Bonnet syndrome (CBS) is characterized by any type of visual hallucination with insight that occurs secondary to sensory deprivation caused by vision loss (1, 2). A misunderstanding of CBS can cause for misdiagnosis by clinicians, with some patients, historically, being admitted to psychiatric units (3) under the assumption that hallucinations are associated with psychological disorders. Recent decades have advanced our understanding of the neurobiological changes that are associated with patients experiencing hallucinations, providing insight for both clinicians and patients that aids appropriate diagnosis and clinical advice. Given that the experience of hallucinations can often be associated with social stigma, understanding the mechanisms of disease had the potential to transform both treatment outcomes and patient experience.

Several theories have been proposed as causes for CBS and these have been detailed elsewhere (1–4). For example, alterations in the short range connectivity between visual processing areas in the brain is known as the hodological theory. Bayesian theories may suggest that prior memory can affect the specific content of the perception (5). One mechanism that has gained substantial support is the deafferentation-hyperexcitability model (6). This suggests that loss of input projections to visual cortex disrupts the excitation-inhibition balance, resulting in increased excitation in some people (7, 8). Bursts of hyper-excitability may then cause an internally generated percept (i.e., a hallucination) to emerge reflecting a change in underlying brain state. This theory captures a potential mechanism to explain how persistent damage can lead to fluctuations in experienced symptoms. This may explain why people experience hallucinations at different times. However, it has been challenging to identify the specific change in neural dynamics that predicts the onset of single hallucination events. This is the part of the mechanism we are interested in exploring.

For example, a recent MRI investigation did not find any neural changes between patients who do and do not experience CBS hallucinations (7). Both the GABA and glutamate levels representing the inhibitory and excitatory neurotransmitters, respectively, were no different between low vision patients with CBS and low vision patients without CBS. Within the CBS patients, there was no correlation between the hallucination score and the GABA concentration. They do not find any structural differences between the two groups. The authors highlight the possibility that their focus on “baseline” measurements may lack sensitivity to more dynamic features of neural activity, which could potentially predict when hallucination onset would be most likely.

Electroencephalogram (EEG) is a great tool for assessing cortical activity, both at rest and dynamically in multiple brain areas (9). Neural dynamics in the human brain can be reflected in the relative power in the canonical frequency bands. For example, “alpha” band activity falls at approximately 8–12 Hz and has been associated with perceptual performance variability (37–39), spatial attention (40–42) and a myriad of other perceptual-cognitive functions (43). Variations in alpha power may indicate inhibitory states (44), or brief periods of pulsed inhibition (45). Induced visual hallucinations have caused changes in EEG alpha and theta activity. This supports the deafferentation model for the formation hallucinations but may also indicate an attentional input in the mechanism (9). A case study using EEG in a single patient with complex visual hallucinations was able to capture hallucination-related changes in neural dynamics, indexed by alpha and theta oscillations (10). Alpha band changes occurred primarily in the occipital region, whereas the theta band changes occurred in the frontal areas. This suggests that noninvasive measures of large-scale brain dynamics could provide an insight into the transient brain states associated with hallucination experiences.

In order to investigate the large-scale neural dynamics associated with hallucination onset, we used EEG recordings and focused on the time period around the onset of individually reported hallucinations. We identified individuals with frequent hallucinations in order to understand dynamic changes related to the onset and offset of the hallucinations using EEG and related this to the hallucinatory experience during the recording session. Given the strong association between alpha band activity and control of visual function, we focused our analyses on changes in alpha power associated with the onset of hallucination periods.

## 2 Materials and methods

### 2.1 Participants

Written informed consent was obtained by all participants and the study received ethical approval by the School Research Ethics Panel (PSY-S21-007) at Anglia Ruskin University. This work adhered to the Declaration of Helsinki. Ten participants were recruited through Esme’s Umbrella and social media. Participants with current active CBS hallucinations, and no other potential source of hallucinations were included as determined from taking a medical history. We sought participants with frequent hallucinations who were likely to experience a hallucination during the recording session. Four participants were excluded. One participant could not clearly delineate when their hallucinations started and stopped as the hallucinations were too frequent, so the data was excluded. There was a technical failure for the other two participants, and the button presses did not register the start and end of the hallucinations so the EEG recordings were not usable in the final analysis. The fourth dataset did not register the time markers accurately due to a software problem. The final six datasets were included. The age range of participants was 37–94 years. A range of visual diseases and degree of visual loss was represented.

### 2.2 Questionnaires

In order to check eligibility for the study, phone screening was undertaken. As part of this, the participants undertook the University of Miami Parkinson’s disease Hallucinations Questionnaire (UM-PDHQ) in order to confirm the presence for CBS and check hallucination frequency (11). Presence of pre-existing ophthalmic disease was confirmed. Cognitive disease was excluded through the use of the Montreal Cognitive Assessment for Blind version (MOCA-BLIND) with low scores (<17) being excluded (12, 13) except in the case of one participant who had a reduced score due to developmental problems since childhood rather than acquired cognitive decline.

Following the EEG session, participants were asked to complete the North East Visual Hallucination Inventory (NEVHI) to detail the hallucinations they experienced over the scan session in detail (14, 15). They were asked to detail each hallucination separately, providing details about its makeup, how long it lasted, how often it occurred, and how much distress it caused. This question session was audio recorded, transcribed, and then subsequently analysed. The hallucination score for the EEG session was calculated as frequency multiplied by duration for every hallucination reported during the EEG session as previously described (7). Full details of the hallucinations experienced during the recording session are provided in Supplementary Table 1.

### 2.3 EEG recording session

Prior to starting the EEG recording session, best corrected visual acuity (BCVA) was recorded with the latest distance correction in place using the Freiburg Vision Test (FrACT) (16). Following this, patients were seated comfortably in a chair in a Faraday cage. Lighting was set to be dim or at a level that would encourage hallucinations. Patients were not constrained in where to look but were encouraged to keep their eyes open. A 32-channel EEG head cap in a 10–20 arrangement was placed on their head (17). EEG was recorded using BrainVision analyzer (BrainVision Analyzer, Version 2.2.2, Brain products GmbH, Gliching, Germany). Electrode impedances were kept below 20 kΩ throughout the recording with a sampling rate of 1,000 Hz for signals. EEG data collection lasted for approximately one hour, during which patients were instructed to press a button for the start and stop of every hallucination experienced during the session. Participants were encouraged to keep their eyes open to prevent them falling asleep. This was monitored using CCTV cameras.

### 2.4 EEG processing

EEG data was pre-processed with EEGLAB (18). Data was epoched 10 s before onset of each hallucination (first button press) and 10 s after the offset of the hallucination (second button press to mark end of hallucination) to mark time before hallucination, time during hallucination, and time after the hallucination. The EEG had a bandpass of 0.5 to 40 Hz. During the recording session, recording continued until the participant experienced at least five hallucinations, up to a maximum recording time of 1 h 15 min. Independent component analysis (ICA) was used to remove eyeblink and eye movement artefacts. Data was referenced to the average from across all channels and the final epochs were exacted for analysis. The analysis epochs were 4,000 ms in duration, centred around the button press events. Each button press was labelled as either “Onset” (i.e., a button press to indicate the onset of a hallucination) or “Offset” (i.e., to indicate the end of a hallucination).

### 2.5 Analysis

#### 2.5.1 Hallucination characteristics

For each participant, we calculated the total number of hallucinations along with the median duration. Additionally, we calculated a total hallucination time measure, corresponding to the sum of the duration of all hallucination periods during the EEG recording session for each individual derived from the button presses. To test whether there were any relationships between these summary descriptive features, we calculated two paired comparisons using Spearman’s rank correlation: (1) median duration vs. number of hallucinations, (2) total hallucination time vs. number of hallucinations.

#### 2.5.2 EEG time-frequency decomposition

To measure changes in alpha band activity, we used EEGLAB *newtimef.m* function to calculate the time-frequency representation. Wavelet cycles were set to increase with a frequency starting at 3 Hz and linearly increasing with frequency to a maximum of 12.8 Hz at the highest. The output were estimates across 23 frequencies (from 5.8–50 Hz) and 200 time points (−1,715 ms: 1,714 ms). Data were baseline-corrected from −1,000 s to −500 ms before the button press response, for both Onset and Offset epochs. Alpha activity was defined as the mean power between 8 and 12 Hz, calculated from electrode Oz. Two time windows of interest were defined within each epoch type: “Pre,” which corresponds to the 500 ms before the button press, and “Post,” which is the 1,500 ms after the button press. To compare the amplitude of alpha, mean power in the Pre and Post windows were compared with a paired *t*-test. To compare alpha dynamics between Onset and Offset epochs, we calculated a difference score: (Onset/Post − Offset/Post) − (Onset/Pre − Offset/Pre). We then used a one-sample *t*-test against zero to test for a significant difference in alpha dynamics. We additionally calculated the event related spectral perturbation (ERSP) for three longer time windows: pre-hallucination (−9 s to −1 s prior to onset button press), during the hallucination and post-hallucination (1 s post to 10 s post hallucination offset response). The ERSP was calculated for each hallucination period and then averaged within a participant to give a single measure of alpha power. No baseline period was used for this analysis. Power was compared between these three epochs with paired *t*-tests.

Control analyses with theta and beta bands followed the same procedure, with theta defined as under 7 Hz and beta activity as 15–30 Hz. For tests of spatial specificity, we used electrode Fz to measure frontal alpha and electrode C3 to capture motor cortical activity. All analysis was non-parametric.

Peak alpha frequency was calculated for all participants using Matlab’s *fft.m*. Data from the pre-hallucination period was used, from −7,000: −1,000 ms prior to button press to onset. Outputs from *fft* were converted to PSD (power spectral density) prior to analysis. Peak alpha frequency was taken as the maximal value within a set range—either 8–12 Hz (“canonical alpha”) or 5–15 Hz [after (19)].

## 3 Results

Patient demographics are shown in Table 1.

**Table 1:** Demographic and screening data for all the participants.

Participant study number	Age	Sex	Eye disease	MOCA score (max 22)	Visual acuity R/L LogMAR	Hallucination score
CBS002	39	F	Complex uveitis	22	LP/LP	4.10
CBS006	65	F	Retinitis pigmentosa	19	NLP/NLP	1.45
CBS007	74	F		17	HM/0.65	7.65
CBS008	67	F	Choroidal melanoma one eye	19	−0.07/artificial eye	0.15
CBS009	37	F	Traumatic injury	18	0.20/0.02	15.00
CBS010	94	M		12^a^	NLP/0.34	1.00

R, right eye; L, left eye; NLP, no light perception; LP, light perception; HM, hand movements. Where diagnosis data was not available it is not listed.

a Low due to developmental cognitive difficulties that have not changed over time. Not related to acquired cognitive reduction.

**Figure 1:**
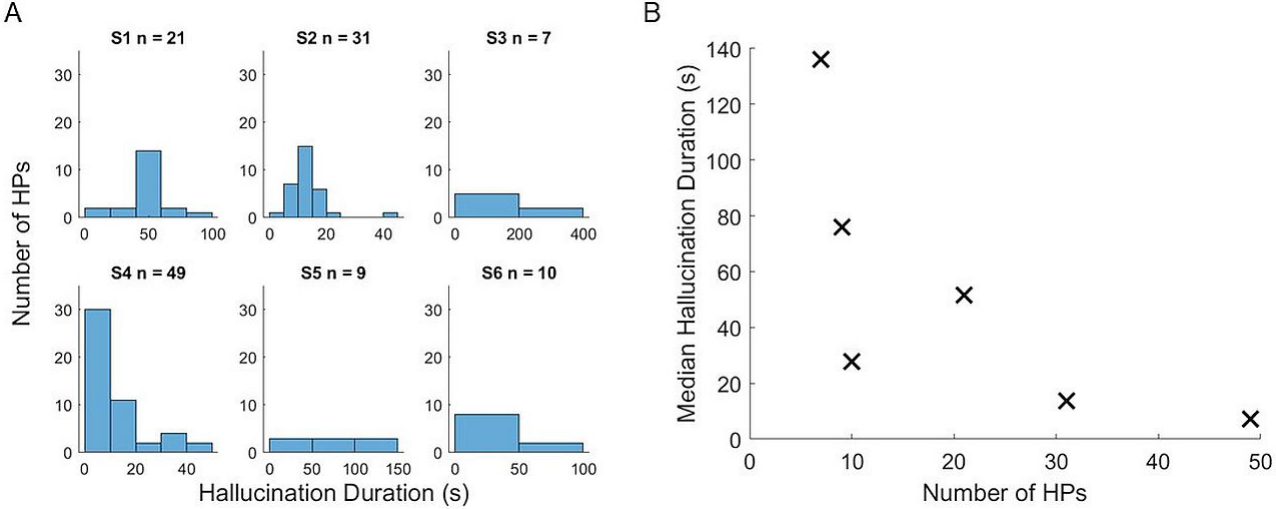
**(A)** Histograms of hallucination period duration for each participant. HP, hallucination period. Individual plots show data from single participants (S1–S6). *X*-axis shows the hallucination episode duration bins. *Y*-axis shows the number of periods per bin. **(B)** Scatter plot shows number of periods (*x*-axis) and the median episodes duration in seconds (*y*-axis). Each point represents data from a single participant.

On average, participants reported 21 hallucinations (range: from 7 to 49). The median duration of individual hallucinations varied both across participants and within participants (see Figure 1), with an overall median duration of 39.6 s. The full details of the hallucinations are detailed in the Supplementary material.

There did appear to be a relationship between duration of reported hallucinations and overall number of hallucinations (Figure 1), with a significant negative correlation observed [*r*(4) = −0.94, *p* = 0.017], such that participants who reported longer hallucination periods also reported fewer overall hallucinations in the recording period. Given the between-participant variability in average hallucination duration, we next tested whether some participants reported overall more time spent experiencing hallucinations. However, there was no relationship between the total reported hallucination time (i.e., the sum of all time between reported onset and offset of hallucinations) and number of reported hallucinations [*r*(4) = −0.49, *p* = 0.36].

Next, we tested our key hypothesis regarding a relationship between changing alpha band dynamics and the onset of reported hallucinations. We focused our analysis on two epochs at the start (“Onset”) and end (“Offset”) of the reported periods of hallucination. Both the Onset and Offset epochs were centred around the button press response (“Pre” and “Post”), which allowed us to take into account any impact of the motor response on alpha band activity. In the Onset epoch, we found a significant difference in alpha power between the Pre and Post time windows [*t*(5) = 4.8, *p* = 0.005]. No significant difference was observed between Pre and Post in the Offset epochs [*t*(5) = 2.0, *p* = 0.10]. This suggests that the period immediately preceding the reported hallucination is associated with lower alpha power than any other time period (Supplementary Figure 1). To formally compare the Onset and Offset periods, we calculated the change in alpha power from Pre to Post button press, and compared the resulting difference score with a repeated measures test, which showed a significant difference [*t*(5) = 8.0, *p* < 0.001]. This suggests that the onset of a visual hallucination was uniquely associated with changes in alpha power.

To investigate any persistent change in alpha band power, we calculated mean power throughout each hallucination period, in additional to extended windows in pre-Onset and post-Offset. This analysis showed significantly lower alpha power in the pre-Onset window, compared to the hallucination period [*t*(5) = 2.7, *p* = 0.045] and a lower alpha power in the pre-Onset vs. post-Offset longer windows [*t*(5) = 2.9, *p* = 0.034]. There was no difference in alpha power between the hallucination period and the post-Offset window [*t*(5) = 1.4, *p* = 0.21], suggesting that low pre-hallucination power is a feature that emerges in neural dynamics over a longer timescale and appears to be a feature that captures a change in brain state co-occurring with hallucination onset.

**Figure 2:**
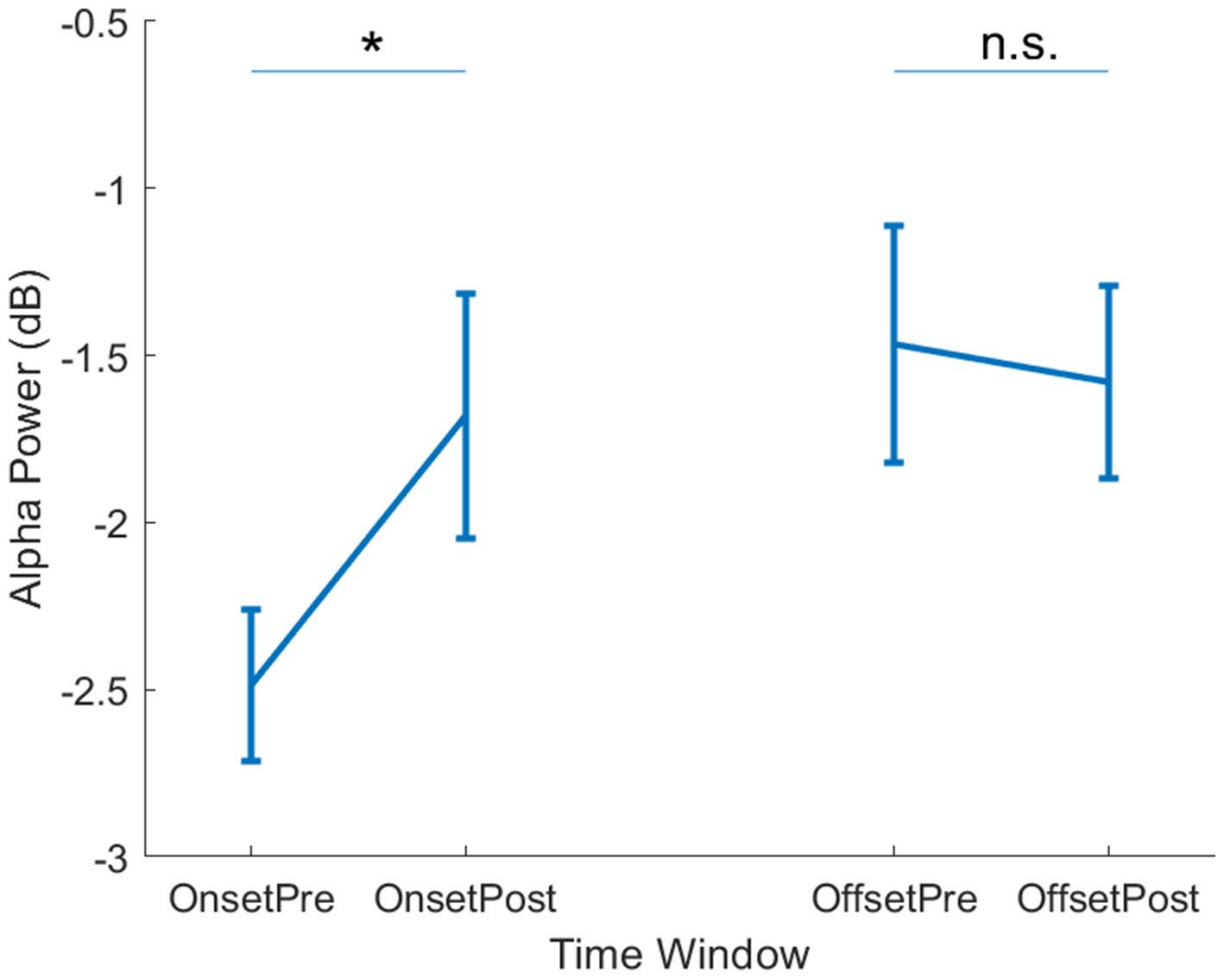
Alpha power per epoch type. Mean alpha power is shown for the Onset (left side) and Offset (right side) epochs. Within each epoch, mean power is shown for the Pre- and Post- button press time windows. Error bars show ±1 SEM. * = *p* < 0.05, n.s. = not significant.

We also calculated the peak alpha frequency from the period just prior to hallucination onset (Supplementary Figure 2) for each participant, given the previously reported reduction in peak alpha for patients with complex hallucinations (19). In our sample, the peak frequency within the 8–12 Hz range was 9.52 Hz. Using a wider window of 5–15 Hz, the peak frequency was 8.4 Hz, which suggests that, for some patients, their neural data were not well captured by a single peak in a PSD representation and lower frequencies may be more prominent than typically expected (see Figure 2).

We therefore tested whether the observed onset-linked dynamics were limited to the canonical alpha band activity by performing a parallel analysis for lower frequency (<8 Hz) and beta band (15–30 Hz) activity. No differences were observed at either onset or offset for theta [Onset: *t*(5) = 0.54, *p* = 0.61; Offset: *t*(5) = 0.089, *p* = 0.93] or beta [Onset: *t*(5) = 2.46, *p* = 0.057; Offset: *t*(5) = 0.074, *p* = 0.94]. This suggests that multiple changes in neural dynamics can be observed at hallucination onset and offset, but only the alpha band activity shows specific change at hallucination onset.

Finally, we investigated the spatial specificity of the alpha band activity by testing for change in alpha power at hallucination onset at prefrontal electrodes, a site of control mechanisms across sensory domains (46). However, there was no change in alpha power across the onset [*t*(5) = 0.018, *p* = 0.99] or offset [*t*(5) = 0.89, *p* = 0.41] of hallucination epochs. Additionally, we saw no change in alpha power at left central electrodes, which should be sensitive to any motor cortical signals, for either onset [*t*(5) = 1.5, *p* = 0.19] or offset [*t*(5) = 0.43, *p* = 0.68]. We therefore only observed changes in alpha dynamics at posterior sites at the onset of self-reported hallucination periods.

## 4 Discussion

This study aimed to establish whether the large-scale dynamics measured by EEG could capture changes in brain state associated with the onset of visual hallucination in CBS patients. We report that there was a decrease in α power specifically related to the onset of CBS hallucinations. As this did not occur when the button is pressed at the end of the hallucination, it is not a motor response related to pressing the button. Alpha oscillations are thought to be critical in information processing via inhibitory control and are particularly prominent over the posterior brain (20). Reduced α power is typically associated with improved visual function (21–23), although this may be dependent on the specific task demands (24). In this study, endogenous fluctuations in alpha power over time may reflect dynamic changes in cortical excitability. During periods of higher excitability, neural populations already subject to decreased inhibitory regulation may be more likely to activate, resulting in the experience of hallucinations.

Our results therefore provide initial insight into a potential mechanism underlying the timing of hallucination onset. This approach highlights the brain states that are associated with spontaneous hallucination onset and is complementary to “trait” approaches, which have characterized the group level differences between patients experiencing hallucinations and those without such experiences. Interestingly, changes in alpha power have also been reported from this perspective (19), suggesting that alpha oscillations could be a “final common pathway” for CBS symptoms. However, other reports have highlighted power change in non-alpha ranges: θ power differences were found to be significant in addition to α power changes in the CBS population compared to a low vision control population (19). Similarly, Yildiz et al. (25) found widespread EEG power changes in patients with CBS compared with low vision patients without CBS in a cross-sectional study. In addition to EEG, fMRI was used to detect changes in occipital activity prior to the onset of the hallucinations in CBS, consistent with our state-based explanation of our present data (26).

Although the present data highlight dynamic alpha power fluctuations as an indicator of hallucination onset, it is unclear from the present data whether there is a causal relationship between alpha power change and hallucination onset. While lower alpha power may increase the probability of a hallucination, alpha power decreases are also observed in response to the onset of visual stimulus onset (event related desynchronization; ERD) (27). In this latter view, alpha power would be an indicator of hallucination onset, rather than a causal mechanism for triggering a hallucination. Future work could therefore use an intervention approach (28) to test the causal relevance of alpha oscillations to hallucination likelihood. daSilva Morgan et al. (14) investigated transcranial direct current stimulation (TDS) for the treatment of CBS. They reported decreases in delta power, theta-alpha ratio, and pre-alpha power following TDS, in addition to a significant decrease in hallucination frequency. An alternative approach could be to use rhythmic entrainment tuned to the individual peak alpha frequency (47), particularly given that the peak alpha frequency appears be lower, or less well defined, in CBS patients, indicating a large-scale alteration to alpha generator mechanisms (19).

The presence of this change in signal could be a marker of a visual percept generated in the visual cortex as the basis of the CBS hallucination. This is critical when put together with other similar work, in order to address knowledge gap observed in some healthcare practitioners (29, 30). This type of mischaracterization of unfamiliar symptoms is not a new phenomenon in the history of medicine. Patients with epilepsy experienced a similar situation. Until we understand more about the biological mechanisms responsible for hallucinations following vision loss, CBS patients remain at risk of incorrect diagnosis and therefore ineffective treatment. For example, it was not until the late 1800s that epilepsy was stopped being treated in a religious and/or superstitious way. However, greater understanding of the biological mechanisms of epilepsy has transformed both diagnoses and patient outcomes (31). In particular, clinicians can measure the presence (or absence) of relevant biomarkers to aid the diagnostic process. People with epilepsy have been subject to much social stigma due to the misconception about the cause (32). The same effects can be seen in chronic pain (33), schizophrenia (34, 35), and many other medical conditions. Finding a change in brain state that appears to be consistent across studies will help with breaking down barriers for both the diagnostic process and suggest fruitful avenues for treatment. The identification of biomarkers will allow CBS to be taken seriously and the wider medical community to be educated on how to diagnose and treat patients appropriately in the same way epilepsy and other conditions have been once their biomarkers have been discovered.

It would be interesting for future work to test the specificity of the α power-hallucination relationship across individuals who experience different types of hallucination (e.g., auditory vs. visual), or who experience greater or lesser insight into the false nature of their hallucinations. These distinctions will help to further improve our understanding of the specific set of biological mechanisms that lead to CBS, opening further opportunities for diagnostics and treatment.

As EEG α waves are not synonymous with consciousness (36), the images cannot be generated at will, so participants would struggle to be aware what is internally generated and what is externally generated. Further work is needed to understand why people experiencing CBS hallucinations retain insight whereas for some other people (e.g., people with psychosis) who experience hallucinations, insight is lost.

Furthermore, we report here additional analyses of the temporal dynamics of hallucination experiences. We found a relationship between number and duration of hallucination, such that patients with longer hallucinations reported fewer periods of hallucination. This is perhaps unsurprising, given our relatively limited period of data recorded. However, we did not find that patients who reported more individual hallucinations experienced, in total, a longer hallucination time within the study. Could indicate that some participants experienced more disjointed periods of hallucinations, while other participants reported a more continuous experience. This could be due to genuinely different experiences, or it could be differences in response criteria—some patients may wait longer before reporting a hallucination has ended. This could be an interesting feature to explore in future work, alongside the strength and specific content of individual hallucinations, all features that contribute to significant individual differences in the experience of CBS patients. The types of hallucinations seen are also highly variable between people. This confirms that each person’s experience of CBS is highly individual.”

This study had a small study sample due to difficulties with recruitment following the COVID-19 pandemic, patients were reluctant to attend on site assessments. We also found it challenging to find people with frequent enough hallucinations to ensure they would hallucinate during the one-hour EEG recording session. The strength of this study is that each participant was their own control participant by providing pre- and post- hallucination data. This pilot study therefore provides a good basis for a larger scale future study on studying dynamic changes in the EEG power changes.

This is an important study in contributing to the jigsaw of understanding an understudied question about changes in the neural dynamics at different timescales. We show a repeatable marker of brain activity changes that occur either at the onset or just prior to the onset of the hallucination. Due to the delays in registering a button press it is not possible to tease apart the exact difference between these points and future work is required to answer the exact question of timing.

## Data Availability

The raw data supporting the conclusions of this article will be made available by the authors on reasonable request.

## Supplementary material

SUPPLEMENTARY FIGURE 1 Time frequency plot showing power within the Onset **(A)** and Offset **(B)** epochs. Zero indicated the time of hallucination onset, indicated by button press response. Plots are averaged across participants. Boxes indicate data included in analysis. Solid box covers the pre- window, dashed line shows post-window.

SUPPLEMENTARY FIGURE 2 Group mean power spectrum. The *x*-axis shows frequency and *y*-axis shows mean amplitude. Error bars (shaded area) shows ±1 SEM.

## References

[1] Baffour-Awuah KA Bridge H Engward H MacKinnon RC Ip IB Jolly JK. The missing pieces: an investigation into the parallels between Charles Bonnet, phantom limb and tinnitus syndromes. Ther Adv Ophthalmol. (2024) 16:25158414241302065. doi: 10.1177/25158414241302065 39649951 PMC11624543

[2] Baffour-Awuah KA Taylor LJ Josan AS Jolly JK MacLaren RE. Investigating the impact of asymmetric macular sensitivity on visual acuity chart reading in choroideremia. Ophthalmic Physiol Opt. (2024) 44:1188–201. doi: 10.1111/opo.13356, 38989810

[3] Adachi N Nagayama M Anami K Arima K Matsuda H. Asymmetrical blood flow in the temporal lobe in the Charles Bonnet syndrome: serial neuroimaging study. Behav Neurol. (1994) 7:97–9. doi: 10.3233/BEN-1994-7209 24487295

[4] Carpenter K Jolly JK Bridge H. The elephant in the room: understanding the pathogenesis of Charles Bonnet syndrome. Ophthalmic Physiol Opt. (2019) 39:414–21. doi: 10.1111/opo.12645, 31591762

[5] Collerton D Barnes J Diederich NJ Dudley R Ffytche D Friston K . Understanding visual hallucinations: a new synthesis. Neurosci Biobehav Rev. (2023) 150:105208. doi: 10.1016/j.neubiorev.2023.105208, 37141962

[6] Reichert DP Seriès P Storkey AJ. Charles Bonnet syndrome: evidence for a generative model in the cortex? PLoS Comput Biol. (2013) 9:e1003134. doi: 10.1371/journal.pcbi.1003134, 23874177 PMC3715531

[7] Bridge H Wyllie A Kay A Rand B Starling L Millington-Truby RS . Neurochemistry and functional connectivity in the brain of people with Charles Bonnet syndrome. Ther Adv Ophthalmol. (2024) 16:25158414241280201. doi: 10.1177/25158414241280201, 39416975 PMC11481065

[8] Burke W. The neural basis of Charles Bonnet hallucinations: a hypothesis. J Neurol Neurosurg Psychiatry. (2002) 73:535–41. doi: 10.1136/jnnp.73.5.535, 12397147 PMC1738134

[9] daSilva Morgan K Elder GJ Ffytche DH Collerton D Taylor J-P. The utility and application of electrophysiological methods in the study of visual hallucinations. Clin Neurophysiol. (2018) 129:2361–71. doi: 10.1016/j.clinph.2018.08.019, 30253375

[10] Piarulli A Annen J Kupers R Laureys S Martial C. High-density EEG in a Charles Bonnet syndrome patient during and without visual hallucinations: a case-report study. Cells. (2021) 10:1991. doi: 10.3390/cells10081991, 34440760 PMC8392863

[11] Papapetropoulos S Katzen H Schrag A Singer C Scanlon BK Nation D . A questionnaire-based (UM-PDHQ) study of hallucinations in Parkinson’s disease. BMC Neurol. (2008) 8:21. doi: 10.1186/1471-2377-8-21, 18570642 PMC2443167

[12] Nasreddine ZS Phillips NA Bédirian V Charbonneau S Whitehead V Collin I . The Montreal Cognitive Assessment, MoCA: a brief screening tool for mild cognitive impairment. J Am Geriatr Soc. (2005) 53:695–9. doi: 10.1111/j.1532-5415.2005.53221.x, 15817019

[13] Wittich W Phillips N Nasreddine ZS Chertkow H. Sensitivity and specificity of the Montreal cognitive assessment modified for individuals who are visually impaired. J Vis Impair Blind. (2010) 6:360–8. doi: 10.1177/0145482X1010400

[14] daSilva Morgan K Schumacher J Collerton D Colloby S Elder GJ Olsen K . Transcranial direct current stimulation in the treatment of visual hallucinations in Charles Bonnet syndrome. Ophthalmology. (2022) 129:1368–79. doi: 10.1016/j.ophtha.2022.06.041, 35817197

[15] Mosimann UP Collerton D Dudley R Meyer TD Graham G Dean JL . A semi-structured interview to assess visual hallucinations in older people. Int J Geriatr Psychiatry. (2008) 23:712–8. doi: 10.1002/gps.1965, 18181237

[16] Bach M. The Freiburg visual acuity test-automatic measurement of visual acuity. Optom Vis Sci. (1996) 73:49–53. doi: 10.1097/00006324-199601000-00008, 8867682

[17] Beniczky S Schomer DL. Electroencephalography: basic biophysical and technological aspects important for clinical applications. Epileptic Disord. (2020) 22:697–715. doi: 10.1684/epd.2020.1217, 33270023

[18] Delorme A Makeig S. EEGLAB: an open source toolbox for analysis of single-trial EEG dynamics including independent component analysis. J Neurosci Methods. (2004) 134:9–21. doi: 10.1016/j.jneumeth.2003.10.009, 15102499

[19] daSilva Morgan K Collerton D Firbank MJ Schumacher J Ffytche DH Taylor JP. Visual cortical activity in Charles Bonnet syndrome: testing the deafferentation hypothesis. J Neurol. (2025) 272:199. doi: 10.1007/s00415-024-12741-2, 39932561 PMC11813974

[20] Klimesch W. Alpha-band oscillations, attention, and controlled access to stored information. Trends Cogn Sci. (2012) 16:606–17. doi: 10.1016/j.tics.2012.10.007, 23141428 PMC3507158

[21] Benwell CSY Tagliabue CF Veniero D Cecere R Savazzi S Thut G. Prestimulus EEG power predicts conscious awareness but not objective visual performance. eNeuro. (2017) 4:ENEURO.0182-17.2017. doi: 10.1523/ENEURO.0182-17.2017, 29255794 PMC5732016

[22] Krasich K Simmons C O’Neill K Giattino CM De Brigard F Sinnott-Armstrong W . Prestimulus oscillatory brain activity interacts with evoked recurrent processing to facilitate conscious visual perception. Sci Rep. (2022) 12:22126. doi: 10.1038/s41598-022-25720-2, 36550141 PMC9780344

[23] Michail G Toran Jenner L Keil J. Prestimulus alpha power but not phase influences visual discrimination of long-duration visual stimuli. Eur J Neurosci. (2022) 55:3141–53. doi: 10.1111/ejn.15169, 33666291

[24] Haarlem CS Mitchell KJ Jackson AL O’Connell RG. Individual peak alpha frequency correlates with visual temporal resolution, but only under specific task conditions. Eur J Neurosci. (2024) 60:5591–604. doi: 10.1111/ejn.16519 39180268

[25] Yildiz S Yulug B Kocabora MS Hanoglu L. Power spectral density and coherence analysis of eye disease with and without visual hallucination. Neurosci Lett. (2021) 740:135444. doi: 10.1016/j.neulet.2020.135444, 33127444

[26] Ffytche DH Howard RJ Brammer MJ David A Woodruff P Williams S. The anatomy of conscious vision: an fMRI study of visual hallucinations. Nat Neurosci. (1998) 1:738–42. doi: 10.1038/3738, 10196592

[27] Pfurtscheller G Aranibar A. Event-related cortical desynchronization detected by power measurements of scalp EEG. Electroencephalogr Clin Neurophysiol. (1977) 42:817–26. doi: 10.1016/0013-4694(77)90235-8, 67933

[28] Pearson J Chiou R Rogers S Wicken M Heitmann S Ermentrout B. Sensory dynamics of visual hallucinations in the normal population. eLife. (2016):e17072. doi: 10.7554/eLife.17072.001 27726845 PMC5059140

[29] Gordon KD Felfeli T. Family physician awareness of Charles Bonnet syndrome. Fam Pract. (2018) 35:595–8. doi: 10.1093/fampra/cmy006, 29471318

[30] Ucan Gunduz G Yalcinbayir O Gelisken O. Awareness of Charles Bonnet syndrome among ophthalmologists: a survey study. Int Ophthalmol. (2025) 45:207. doi: 10.1007/s10792-025-03575-6, 40418493

[31] Magiorkinis E Diamantis A Sidiropoulou K Panteliadis C. Highlights in the history of epilepsy: the last 200 years. Epilepsy Res Treat. (2014) 2014:582039. doi: 10.1155/2014/582039, 25210626 PMC4158257

[32] Kaculini CM Tate-Looney AJ Seifi A. The history of epilepsy: from ancient mystery to modern misconception. Cureus. (2021) 13:e13953. doi: 10.7759/cureus.13953, 33880289 PMC8051941

[33] Poisbeau P Salvat E. Neurophysiology of acute and chronic pain: from genes to pain symptoms. BJA Educ. (2025) 25:357–66. doi: 10.1016/j.bjae.2025.04.008, 40842915 PMC12365510

[34] Coyle JT Balu DT Puhl MD Konopaske GT. History of the concept of disconnectivity in schizophrenia. Harv Rev Psychiatry. (2016) 24:80–6. doi: 10.1097/HRP.0000000000000102, 26954593 PMC4788103

[35] Jablensky A. The diagnostic concept of schizophrenia: its history, evolution, and future prospects. Dialogues Clin Neurosci. (2010) 12:271–87. doi: 10.31887/DCNS.2010.12.3/ajablensky, 20954425 PMC3181977

[36] Colombo MA Comanducci A Casarotto S Derchi CC Annen J Viganò A . Beyond alpha power: EEG spatial and spectral gradients robustly stratify disorders of consciousness. Cereb Cortex. (2023) 33:7193–210. doi: 10.1093/cercor/bhad031, 36977648 PMC10233271

[37] Bays BC Visscher KM Le Dantec CC Derchi CC Seitz AR. Alpha-band EEG activity in perceptual learning. J Vis. (2015) 15:7. doi: 10.1167/15.10.7, 26370167 PMC4570730

[38] Cecere R Rees G Romei V. Individual differences in alpha frequency drive crossmodal illusory perception. Current Biology. (2015) 25:231–235., 25544613 10.1016/j.cub.2014.11.034 PMC4300399

[39] Ergenoglu T Demiralp T Bayraktaroglu Z Ergen M Beydagi H Uresin Y . Alpha rhythm of the EEG modulates visual detection performance in humans. Brain Res. Cogn. Brain Res. (2004) 20:376–83. doi: 10.1016/j.cogbrainres.2004.03.009, 15268915

[40] Clayton MS Yeung N Cohen Kadosh R. Electrical stimulation of alpha oscillations stabilizes performance on visual attention tasks. Journal of Experimental Psychology: General, (2019) 148:203–220., 30421943 10.1037/xge0000502

[41] Lu R Michael E Scrivener CL Jackson JB Duncan J Woolgar A . Parietal alpha stimulation causally enhances attentional information coding in evoked and oscillatory activity. Brain Stimul. (2025) 18:114–127. doi: 10.1016/j.brs.2025.01.003, 39778653

[42] Thut G Nietzel A Brandt SA Pascual-Leone A. Alpha-band electroencephalographic activity over occipital cortex indexes visuospatial attention bias and predicts visual target detection. J Neurosci. (2006) 26:9494–502. doi: 10.1523/JNEUROSCI.0875-06.2006, 16971533 PMC6674607

[43] Clayton MS Yeung N Cohen Kadosh R. The many characters of visual alpha oscillations. Eur J Neurosci. (2018) 48:2498–2508. doi: 10.1111/ejn.13747, 29044823

[44] Mazaheri A Jensen O. Rhythmic pulsing: linking ongoing brain activity with evoked responses. Front Hum Neurosci. (2010) 14:177. doi: 10.3389/fnhum.2010.00177, 21060804 PMC2972683

[45] Mathewson KE Lleras A Beck DM Fabiani M Ro T Gratton G . Pulsed out of awareness: EEG alpha oscillations represent a pulsed-inhibition of ongoing cortical processing. Front Psychol. (2011) 19:99. doi: 10.3389/fpsyg.2011.00099, 21779257 PMC3132674

[46] Duncan J Owen AM. Common regions of the human frontal lobe recruited by diverse cognitive demands. Trends Neurosci. (2000) 23:475–83. doi: 10.1016/s0166-2236(00)01633-7, 11006464

[47] Michael E Covarrubias LS Leong V Kourtzi Z. Learning at your brain’s rhythm: individualized entrainment boosts learning for perceptual decisions. Cereb Cortex. (2023) 33:5382–5394. doi: 10.1093/cercor/bhac426, 36352510 PMC10152088

