# Supplementary figures and images for: EEG changes associated with hallucinations caused by Charles Bonnet Syndrome
Source: Front Neurol. 2026 Jan 6;16:1697094. doi: 10.3389/fneur.2025.1697094 (PMC12815872; doi:10.3389/fneur.2025.1697094)

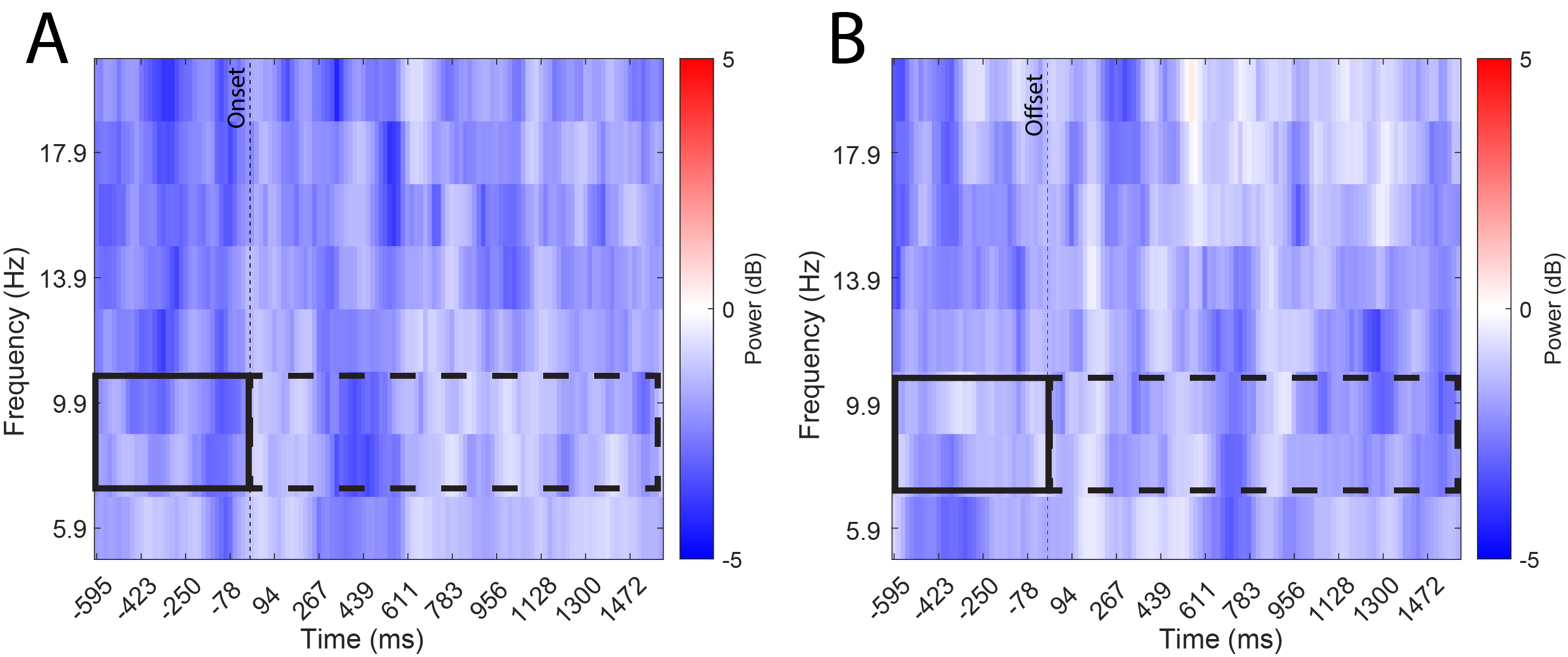

Supplement: SUPPLEMENTARY FIGURE 1 — Time frequency plot showing power within the Onset (A) and Offset (B) epochs. Zero indicated the time of hallucination onset, indicated by button press response. Plots are averaged across participants. Boxes indicate data included in analysis. Solid box covers the pre- window, dashed line shows post-window. [file Image_1.png]

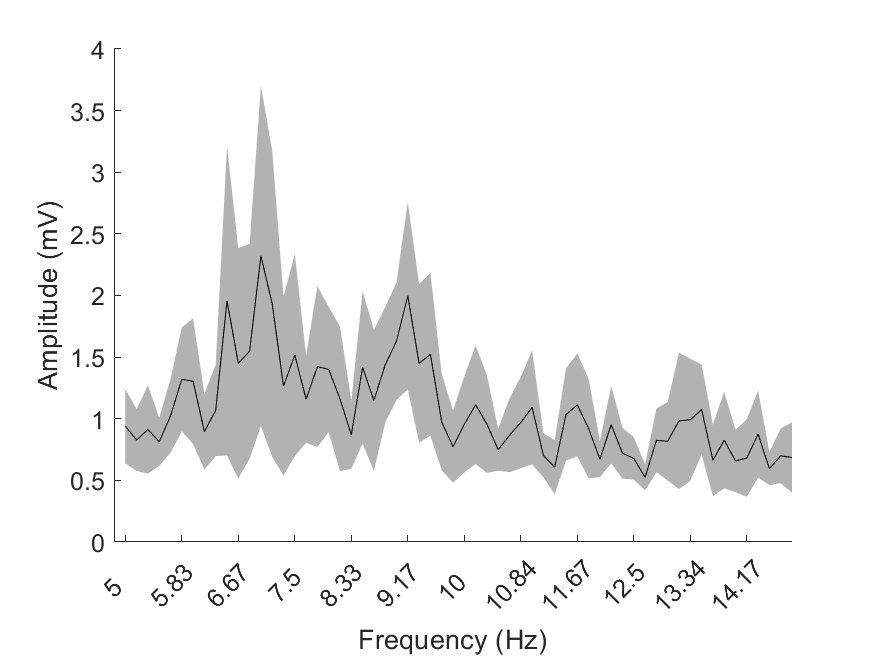

Supplement: SUPPLEMENTARY FIGURE 2 — Group mean power spectrum. The x-axis shows frequency and y-axis shows mean amplitude. Error bars (shaded area) shows ±1 SEM. [file Image_2.png]
