# Supplementary material for: EEG changes associated with hallucinations caused by Charles Bonnet Syndrome
Source: Front Neurol. 2026 Jan 6;16:1697094. doi: 10.3389/fneur.2025.1697094 (PMC12815872; doi:10.3389/fneur.2025.1697094)
Supplement: Supplementary file 3 [file Table_1.docx]

| Participant ID | Description | Number of times for each hallucination | Duration of hallucination | Final Hallucination score incorporating distress |
| --- | --- | --- | --- | --- |
| CBS002 | Participant had 2 types of hallucinations throughout the session. First type: Images of small people similar to cartoon figures that kept staring and smiling. Second hallucination: people dressed in Victorian-like clothing that were looking at him smiling and laughing. | First type:  3 times  Second type:  3 times | Both types lasted for seconds | 7.65 |
| CBS005 | Participant had 4 types of hallucinations. First type was an image of a blue flash that grew bigger. Second type was a curl of white light that disappeared and reappeared. Third type was a kingfisher bird that was black, white, and grey. Fourth type were rhombuses that were red/blue/beige that were wider at the top and flatter at the bottom and moved around and had tales of light coming out of them. | First type: once  Second type: once  Third type: once  Fourth type: 3 times | All types lasted for seconds | 0.15 |
| CBS006 | Participant had 3 types of hallucinations. First type involved 3D dots, like bubble wrap that were transparent and small. Second type were scribbles that were black and blue and they jump all together and can look like insect spiders and cobwebs. Third type involved images of circles and spirals. Spirals moved and had 5 lines coming out of them. | First type: 3 times  Second type: 2 times  Third type: 4 times | All types of hallucination lasted for minutes. | 15 |
| CBS007 | Participant saw one type of hallucination throughout the session. Participant saw images of flower heads that were pinkish in colour. All images were the same throughout the session. | Occurred 10 times | Lasted for seconds | 1 |
| CBS008 | Participant saw 6 types of hallucinations. First type included alien faces with skull-like shape. Second involved images of green light surrounded white light like lava lamp blobs. Third involved images of blobs. Fourth involved images of dim alien faces with glowing eyes that were stationary. Fifth type included images of outlines of people in red with grey line with just the head and shoulders that did not move. Sixth type includes a lot of multicoloured shapes with cartoon-like eyes. | First type: 15 times  Second type: 9 times  Third type: 7 times  Fourth type: 4 times  Fifth type: 12 times  Sixth type: 3 times | All types of hallucinations lasted for seconds except the fourth type which lasted for minutes. | 4.1 |
| CBS009 | Participant saw one type of hallucination throughout the recording session. This included drifts of white lines that would spin clockwise then stop and start again when a camera flash affects the whole visual field. This happened throughout the entire EEG session. | Occurred 22 times. | Lasted for seconds | 2.2 |
| CBS010 | Participant saw two types of hallucinations. The first type included an image of a sofa armchair with faded colours of burnt orange, red cream black and brown. The second type included images of a sea sponge that was creamy yellow coloured and was rectangular, had holes and was textured. | First type: 28 times  Second type: once | both types lasted for seconds | 1.45 |

**Supplementary Table 1.** NEVHI questionnaire results describing the hallucination characteristics from the recording session. *Note.* Higher scores indicate more hallucinations and higher distress.
